# Gypenoside LI modulates autophagy-associated apoptosis in clear cell renal cell carcinoma by PPARG/PTEN/PI3K/AKT/mTOR/ULK1 signaling network

**DOI:** 10.3389/fphar.2026.1956477

**Published:** 2026-10-08

**Authors:** Ruyi Liu, Hao Yu, Hui Liu, Yujie Wei, Anxin Wang, Qi Huang, Hui Xu, Xiuming Li

**Affiliations:** 1 Department of Urology, Affiliated Hospital of Chengde Medical University, Chengde, China; 2 Department of Prescription & TCM, School of Traditional Chinese Medicine, Chengde Medical University, Chengde, China; 3 Hebei Universities Characteristic Sericulture Application Technology Research and Development Center, Sericultural Research Institute, Chengde Medical University, Chengde, China; 4 Hebei Key Laboratory of Panvascular Diseases, Chengde, China

**Keywords:** autophagy, clear cell renal cell carcinoma, gypenoside LI, PPARG, ULK1

## Abstract

**Objective:**

This study was conducted to comprehensively evaluate the anti-clear cell renal cell carcinoma (ccRCC) activity of gypenoside LI (Gyp LI) through *in vitro* and *in vivo* models; elucidate the specific molecular pathways mediating the induction of apoptosis and autophagy in Gyp LI-treated ccRCC cells, focusing on the regulatory role of Gypenoside LI in upregulating PPARG and coordinately modulating the PTEN/PI3K/AKT/mTOR-ULK1 signaling network; and render experimental evidence and theoretical support to develop Gyp LI as a novel anti-ccRCC agent.

**Materials and Methods:**

Human ccRCC cell lines (769-P and ACHN) and a BALB/c nude mouse xenograft model were used for this study. The suppressive effect of Gyp LI on ccRCC cell proliferation was assessed by colony formation assay, Cell Counting Kit-8 (CCK-8) assay, wound healing assay, and Hoechst staining. Autophagy-related ultrastructural changes in treated cells were observed by transmission electron microscopy. Western blot and quantitative real-time PCR (RT-qPCR) were conducted to evaluate fluctuations in the expression levels of autophagic and apoptotic process markers. Putative therapeutic targets associated with Gyp LI were predicted utilizing a network pharmacology framework. Concurrently, the transcriptional control exerted by PPARG over the *PTEN* gene was validated by dual-luciferase reporter systems and chromatin immunoprecipitation combined with quantitative PCR. To clarify the regulatory relationships within the signaling pathway, we performed *in vitro* rescue experiments using target gene knockdown. Additionally, a subcutaneous xenograft model in nude mice was established for assessing the *in vivo* antitumor activity and biosafety of Gyp LI. Apoptosis-, autophagy-, and pathway-related protein expression in tumor tissues was assessed by Western blotting (WB) assay.

**Results:**

CCK-8 reagent-based cell viability assay revealed that Gyp LI dose-dependently inhibited 769-P and ACHN cell growth, with 24-h IC_50_ values of 62.57 and 79.30 μM, respectively. Colony formation, wound healing, and Hoechst staining assay results collectively showed that Gyp LI suppressed cell proliferation and migration and exerted apoptotic effects. Morphological and molecular assays showed that Gyp LI-treated ccRCC cells exhibited prominent autophagosomes and autolysosomes, with upregulation of the autophagy-related proteins Beclin1, ATG5, and LC3 I/II and downregulation of P62. The antiapoptotic factor Bcl-2 exhibited substantially reduced expression, while the proapoptotic mediators caspase-3 and Bax were upregulated, these findings indicated concurrent autophagy-related alterations and apoptosis in Gyp LI-treated ccRCC cells. WB and RT-qPCR assays revealed that Gyp LI upregulated the mRNA and protein expression of the transcription factor PPARG. PTEN transcription was stimulated by the specific binding of PPARG to its promoter region. The resulting intracellular accumulation of PTEN effectively blocked abnormal activation associated with the PI3K/AKT/mTOR signaling axis and ultimately modulated autophagy-associated apoptosis through the phosphorylation-mediated activation of ULK1. ULK1 knockdown reversed the autophagic phenotype modulated by Gyp LI. Moreover, PPARG knockdown reversed Gyp LI-mediated PTEN upregulation, PI3K/AKT/mTOR pathway inhibition, and ULK1 phosphorylation, while attenuating Gyp LI-modulated autophagy and apoptosis. *In vivo* experiments showed that oral administration of Gyp LI significantly inhibited xenograft tumor growth but did not affect body weight in nude mice. WB assay further confirmed increased expression of proapoptotic and autophagy-related proteins and decreased expression of phosphorylated AKT/mTOR pathway proteins in Gyp LI-treated tumor tissues. However, further studies are required to fully elucidate the autophagic flux and the direct binding targets.

**Conclusion:**

This study revealed that Gyp LI promotes cell death associated with autophagic alterations in ccRCC cell lines. Mechanistically, this phenomenon is driven by upregulating PPARG and coordinately modulating the PTEN/PI3K/AKT/mTOR-ULK1 signaling network, but the exact causal interdependencies and comprehensive biosafety profile warrant further investigation before any clinical translation. The *in vivo* efficacy suggests that Gyp LI could serve as promising candidates for plant-derived therapeutic interventions for treating RCC.

## 1 Introduction

Clear cell renal cell carcinoma (ccRCC) is a highly prevalent histological variant among renal tumors and constitutes a significant portion of all renal malignancies. According to the global epidemiological data from the 2022 GLOBOCAN database (IARC), approximately 434,840 new cases of renal cancers and 155,953 renal cancer-related deaths were recorded worldwide. Furthermore, the assessment of age-adjusted metrics indicates a prevalence rate of 4.4 and a mortality ratio of 1.5 per 100,000 individuals, which positions this disease at the 14th and 16th ranks among all oncological conditions, respectively (Motzer et al., 2024).

*Gynostemma pentaphyllum*, a climbing plant of the Cucurbitaceae family, mainly occurs in Guangxi, Yunnan, Hunan, and other regions of China. Gypenosides, primary bioactive saponins isolated from *G. pentaphyllum*, are widely utilized for treating several diseases in traditional Chinese medicine. The antineoplastic effects of gypenosides are mediated through diverse biological pathways such as malignant cell proliferation inhibition, cellular apoptosis induction, and immune response modulation (Liu et al., 2025; Tan et al., 2022; Xiao et al., 2024).

Although numerous saponins have been extensively studied, the existing scientific literature regarding gypenoside LI (Gyp LI) remains preliminary. Specifically, there are no comprehensive investigations of the tumor-suppressive properties of gypenosides in ccRCC models and the related molecular pathways.

As a highly conserved degradation pathway in eukaryotic cells, autophagy depends on the formation of double-membraned autophagosomes (Hurley and Young, 2017). These specialized vesicles sequester damaged organelles, toxic protein aggregates, and invading pathogens for subsequent lysosomal degradation. The subsequent fusion of these vesicles with lysosomes facilitates the degradation of their cargo and enables the recycling of molecular components, a process essential for maintaining metabolic homeostasis and overall cellular health. Under abnormal conditions such as hypoxia, oxidative stress, nutrient deprivation, and pharmacological stimulation, the rapid activation of autophagy enables cellular adaptation to adverse microenvironments and simultaneously ensures adequate supply of essential metabolic substrates. Notably, autophagy plays a complex, context-dependent role in tumorigenesis and tumor progression. On the one hand, it can suppress tumor initiation by mitigating oxidative damage and preserving genomic stability; on the other hand, once tumors are established, autophagy can help cancer cells withstand stress, evade immune clearance, and develop resistance to therapeutic interventions (Vargas et al., 2023).

Dysregulation of autophagy-related pathways, including mitogen-activated protein kinase and PI3K/ AKT /mTOR signaling cascades, is closely associated with RCC progression and treatment resistance (Debnath et al., 2023).

Although previous research has demonstrated that gypenosides can regulate oxidative stress and autophagy in various cancer cells (Pan et al., 2024), their effects on RCC-related molecular networks require further investigations. Here, we utilized the human ccRCC cell lines 769-P and ACHN and a xenograft tumor nude mouse model to systematically evaluate the anti-ccRCC activity of Gyp LI. We further explored the molecular mechanisms through which Gyp LI regulates autophagy and apoptosis in ccRCC cells, with particular focus on Gypenoside LI upregulating PPARG and coordinately modulating the PTEN/PI3K/AKT/mTOR-ULK1 signaling network. These findings may provide valuable experimental evidence for the potential application of Gyp LI as an anti-ccRCC therapeutic agent. A schematic overview of the anti-ccRCC mechanism of Gyp LI investigated in this study is provided (Figure 1).

**FIGURE 1:**
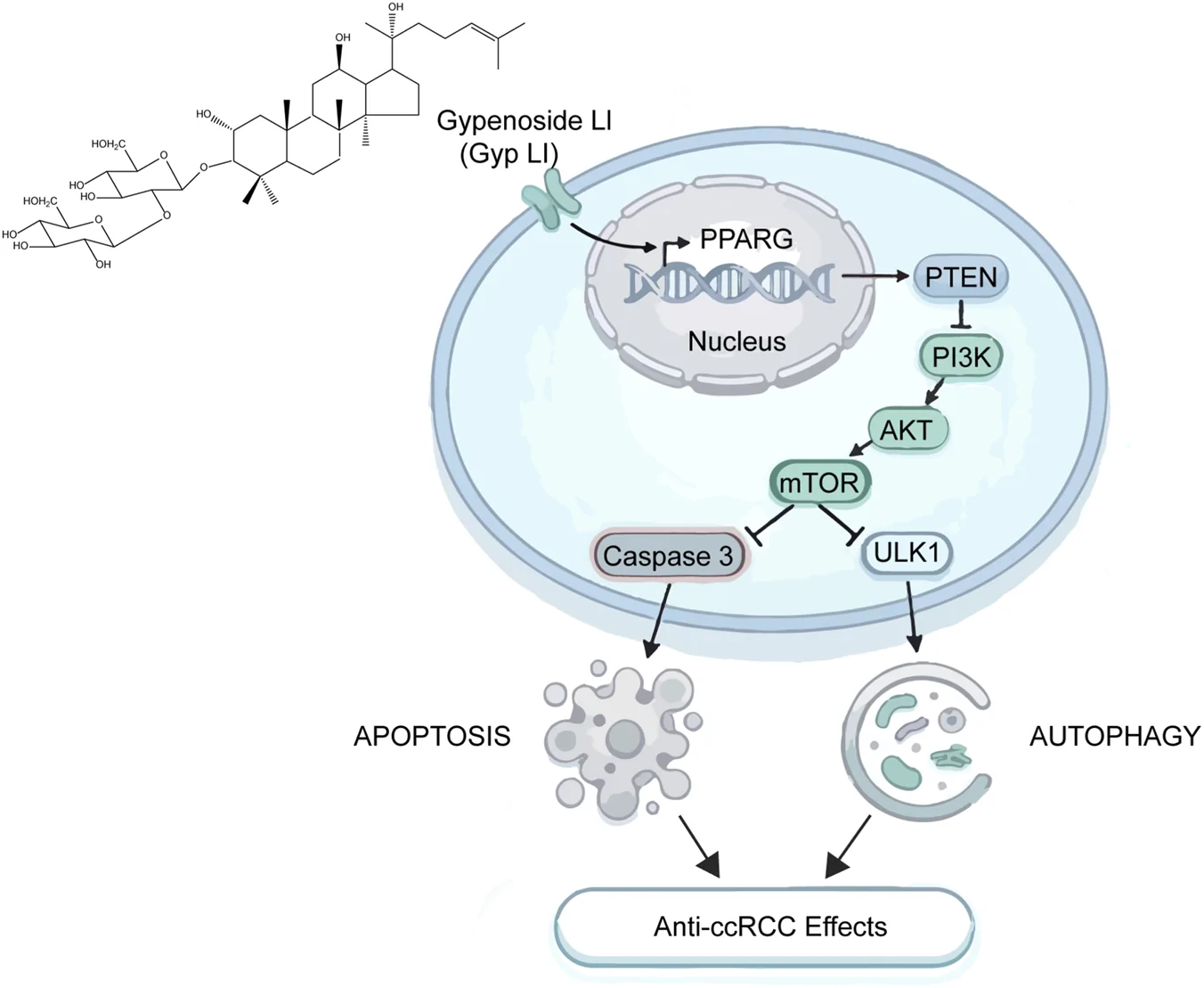
Graphical abstract depicting the anti-ccRCC activity of Gyp LI.

## 2 Materials and methods

### 2.1 Chemical reagents

Gyp LI was extracted and identified according to a previously established protocol (Xing et al., 2018). Its purity was greater than 99%, as confirmed by liquid chromatography-mass spectrometry, and it was stored at −20 °C under light-protected conditions. The following antibodies were used: Bcl-2, Bax, caspase-3, MAP1LC3A, Beclin1, P62, ATG5, AKT, p-AKT (T450), mTOR, p-mTOR (S2448), PI3K, ULK1, Phospho-ULK1(S556), PPARG, PTEN, Ki-67, and β-actin (Cat. Nos. PD01-41, PSHO-20, SP01-02, ST47-03, JE59-31, PS00-61, SN73-07, ST05-09, SD08-12, SU30-00. HA600094, ET1608-70, JA58-36, ET1609-26, ET1606-43, RT1668, ST50-01, and EM21002, respectively; all from Huabio).

### 2.2 Cell culture

769-P and ACHN cell lines were supplied by the cell culture repository of the Chinese Academy of Sciences. The absence of cross-contamination was confirmed by short tandem repeat authentication, and cells from passages 3–10 were used for the experiments. Distinct basal nutritional solutions (Gibco, China) were used for the routine propagation of these cells. Specifically, RPMI 1640 medium and DMEM were employed for culturing 769-P and ACHN cell populations, respectively. Both culture media were supplemented with 10% fetal bovine serum (BioInd) and 1% antibiotic mixture containing penicillin and streptomycin (BasalMedia) to ensure robust cell viability and prevent microbial contamination. All cultures were incubated in a humidified environment supplied with 5% CO_2_ at 37 °C.

### 2.3 Cell viability assay

Cell proliferation assays were performed by seeding 769-P and ACHN cells into 96-well culture plates (density: 10,000 cells/well). After 24 h incubation to ensure uniform surface adherence of the cells, the culture medium was substituted with various concentrations of Gyp LI (0–80 μM), and cell incubation was continued for one additional day. The therapeutic solutions of Gyp LI were freshly obtained by diluting the primary stock (prepared using 20 mM dimethyl sulfoxide (DMSO)) with a nutrient-rich medium, ensuring that the vehicle’s final concentration was not more than 0.1%. To assess the viability of living cells, Cell Counting Kit-8 reagent (CCK-8; Dojindo Laboratories, China; 10 μL) was added to each well, followed by incubation for 90 min to facilitate the reaction process. A FLUOstar Omega spectrophotometer (BMG Labtech) was employed for measuring the optical density at 450 nm.

### 2.4 Colony formation assay

To determine the long-term proliferative abilities of the cell lines, single cell dispersions of 769-P and ACHN cells were seeded into 12-well plates (density: 150 cells/well). After 24 h incubation to facilitate cell adherence, the cells were exposed to the designated Gyp LI treatments. All experimental samples were then incubated at 37 °C for 14 days in a controlled humidified environment with 5% CO_2_. Macroscopic clusters were subsequently immobilized with paraformaldehyde (4% w/v) and stained with 0.1% crystal violet. Enumeration of visible cell groupings containing at least 50 constituent cells was achieved with ImageJ software. The final colony formation ratio was estimated by evaluating the proportion of successfully developed colonies relative to the initially seeded cell population.

### 2.5 Hoechst staining assay

769-P and ACHN cells were exposed for 24 h to different Gyp LI concentrations. After exposure, the cells were rinsed in Buffer A and immobilized by treatment with 4% formaldehyde for 10 min. The nuclear material was stained subsequently with Hoechst 33258 (1:10 dilution; Jiangsu KeyGEN BioTECH Corp., Ltd., China) for 10 min without external heating. Fluorescence emission was quantified by fluorescent microscopy at an excitation frequency of 340 nm.

### 2.6 Wound healing assay

Cells were grown in standard 6-well plates until absolute cell confluency was achieved. A sterile 200-μL pipette tip was used to form a linear scratch wound in the center of cell monolayer. To evaluate cell migration, the culture medium was substituted with Gyp LI-containing serum-free medium. Wound closure was evaluated by acquiring images at 0 and 24 h by phase-contrast microscopy. Wound healing was quantified with ImageJ software, and the wound healing rate was determined as follows [initial wound area − remaining wound area/initial wound area] × 100%.

### 2.7 Western blotting (WB) assay

Total cellular proteins were extracted with RIPA lysis buffer containing protease and phosphatase inhibitors (Beyotime Biotechnology). Protein samples (20 μg) were separated by SDS-PAGE on a 10% gel, and the separated proteins were then transferred onto PVDF membranes (Millipore) by semi-dry electroblotting at 250 mA for 2 h. The membranes were blocked with 5% skim milk and then incubated with primary antibodies overnight at 4 °C. Following incubation with secondary antibodies, target signals were developed using ECL solutions (Thermo Fisher) and quantified by densitometry with Image Lab software v6.0,Bio−Rad .

### 2.8 Transmission electron microscopy (TEM)

Cells were initially cultured in 6-well plates (density: 2.5 × 10^5^ cells/mL), treated with Gyp LI, and prepared for observation by TEM. Following the treatment, the cells were washed thrice with phosphate-buffered saline (PBS), fixed with osmium tetroxide for 2 h, and rinsed again with PBS. The samples were embedded in epoxy resin, polymerized in an oven, and sectioned with an ultramicrotome to obtain ultrathin slices of approximately 50–60 nm thickness. After staining with lead citrate and uranyl acetate, the sections were imaged by TEM.

### 2.9 Network pharmacology and target identification

#### 2.9.1 Identification of Gyp LI- and RCC-related targets

The chemical structure of Gyp LI was constructed using ChemDraw 23.1 and imported into the Swiss Target Prediction platform (http://swisstargetprediction.ch/) for predicting the molecular targets of Gyp LI. RCC-related genes were retrieved from GeneCards, Online Mendelian Inheritance in Man (OMIM), Therapeutic Target Database (TTD), DrugBank, and PharmGKB by using the keywords “RCC,” “Kidney Cancer,” “Renal carcinoma,” “Renal cell carcinoma,” and “Renal tumour,” with the species restricted to humans. A higher score in the GeneCards database indicates a stronger association between a target and the disease. Empirically, when the number of targets was excessive, targets with scores exceeding the median value were classified as potential RCC-related targets. Targets extracted from the five databases were integrated and deduplicated to establish a comprehensive RCC-related target set.

#### 2.9.2 Network construction

Interactions between Gyp LI-associated targets and RCC-related targets were examined by identifying common targets with Venny 2.1.0 (https://bioinfogp.cnb.csic.es/tools/venny/). The STRING 12.0 database (https://string-db.org/) was employed for constructing a protein-protein interaction (PPI) network for the identified common targets, with “*Homo sapiens*” and “highest confidence” (>0.4) as the parameters; all remaining settings were configured as default values. Cytoscape v3.9.1 was utilized for constructing the PPI network, and core targets were identified based on network topology parameters, particularly focusing on the degree value.

### 2.10 Terminal deoxynucleotidyl transferase-mediated dUTP nick-end labeling (TUNEL) assay

Cells were seeded into coverslips and routinely cultured to the appropriate cell density before the indicated treatments. After treatment exposure, the cells were immobilized with ambient 4% paraformaldehyde, subjected to a standardized washing procedure, and permeabilized with ice-cold 0.1% Triton X-100. Apoptotic DNA fragmentation was detected by TUNEL assay as instructed by the manufacturer (Roche Diagnostics, Mannheim, Germany).

### 2.11 Xenograft tumor experiment

Four-to six-week-old, specific pathogen-free (SPF), male BALB/c nude mice (weight: 18–22 g) were supplied by Beijing Vital River Laboratory Animal Technology Co., Ltd (license No. SCXK [Jing] 2023–0,001). During the acclimatization phase and the experimental procedures, mice were housed in the SPF animal facility of Chengde Medical University under standard environmental conditions: 12-h light/dark cycle, food and water *ad libitum*, humidity: 50%–60%, and controlled temperature: 22 °C–25 °C. Prior to study commencement, all experimental procedures were reviewed and approved by the Animal Welfare and Ethics Committee of Chengde Medical University (approval No. CYFYLL2023637). Sixteen nude mice were randomly allocated into four groups (*n* = 4 per group) using a simple randomization method: shCon group, shPPARG group, shPPARG + Gyp LI group, and Gyp LI group. All mice were subcutaneously administered 5 × 10^6^ ACHN cells in the right dorsal region behind the scapula. Tumor volume and body weight were monitored beginning on post-inoculation day 3. Gyp LI was dissolved in a vehicle containing 10% DMSO, 5% Tween-80, 40% polyethylene glycol 300, and 45% normal saline to prepare a 12 mg/mL solution. When tumor volume was 100 mm^3^, intervention group mice received Gyp LI by oral gavage dose:0.1mL/20g once daily for 35 consecutive days. Mice were then euthanized, and their tumor tissues were collected, weighed, and subjected to subsequent immunohistochemical (IHC) analysis. No predefined exclusion criteria were established, and no mice were excluded during the experiment. Due to laboratory constraints, the investigators were not blinded to the group allocation during tumor measurement and outcome assessment. Statistical analysis was performed using a two-way ANOVA.

### 2.12 IHC analysis

Ten-micrometer-thick sections derived from freshly frozen neoplastic tissues were subjected to standard IHC analysis. Following heat-mediated antigen retrieval (citrate buffer [pH 6.0] treatment for 35 min) and quenching of endogenous peroxidase activity (3% H_2_O_2_ treatment for 10 min) sequentially, the samples were saturated for 1 h with 5% bovine serum albumin and then incubated with primary antibodies (Ki-67, caspase-3, Beclin1, Bcl-2 Bax, and ATG5) overnight at 4 °C. Subsequently, the sections were incubated with HRP-labeled secondary antibodies (ZSGB-BIO, China) at 37 °C for 30 min. Color development for target localization was achieved using a DAB substrate system and counterstaining with hematoxylin.

### 2.13 Dual-luciferase reporter assay

Reporter vectors carrying the target sequence were co-transfected into cells with a Renilla luciferase internal control plasmid at the designated ratio. As instructed by the manufacturer, the transfection reagent was used to co-transfect the reporter plasmid, internal control plasmid, and corresponding treatment plasmids into the cells. Following 2 days of transfection, the existing nutrient broth was removed from the culture vessels. Subsequently, the cellular monolayer was subjected to a single rinse with PBS. Finally, a 15-min period of ambient disruption was initiated by introducing a 1× passive lysis buffer. The resulting lysate supernatant was then transferred to a white opaque 96-well plate, and LAR II and Stop and Glo Reagent were sequentially added to measure Firefly luciferase and Renilla luciferase activities, respectively. To address variations in transfection efficiency, the luminescent signals from the Firefly luciferase reporter were standardized against the corresponding emissions from Renilla luciferase.

### 2.14 Chromatin immunoprecipitation (ChIP)-quantitative real-time PCR (qPCR) assay

ChIP combined with qPCR (ChIP-qPCR) was conducted for detecting target protein binding to genomic regions. Briefly, cells were crosslinked with 1% formaldehyde for 10 min at 37 °C; the reaction was then terminated with glycine. The cells were subsequently washed with pre-cooled PMSF-containing PBS, lysed in SDS lysis buffer on ice, and subjected to ultrasonic chromatin fragmentation under low-temperature conditions. After decrosslinking for 4 h at 65 °C, the phenol-chloroform method was used to extract DNA, and chromatin fragmentation efficiency was determined by agarose gel electrophoresis. The ultrasonicated supernatant was diluted with ChIP dilution buffer, and a portion of the sample was reserved as the input control. The remaining sample was pre-cleared with Protein A + G agarose beads to minimize nonspecific binding, followed by overnight incubation with specific primary antibodies at 4 °C. Immunocomplexes were enriched and sequentially washed with low-salt, high-salt, LiCl, and TE buffers. Purified DNA was obtained after proteinase K digestion and column purification and quantified for subsequent qPCR analysis. A 20-μL reaction system was used, and amplification was performed for 40 cycles using a two-step or three-step protocol according to the primer annealing temperature. Finally, qPCR or PCR was conducted to amplify the targeted DNA. ChIP-specific primer sequences are listed in Supplemental Materials.

### 2.15 Construction and validation of shRNA knockdown plasmids

Specific shRNA sequences targeting the human *PPARG* gene and a negative control (shCon) were designed. Complementary single-stranded oligonucleotides were synthesized, denatured at 95 °C, and annealed through a gradual cooling process to form double-stranded DNA. The expression vector backbone pLKO.1-puro was double-digested with restriction enzymes (*AgeI* and *EcoRI*), and the annealed double-stranded DNA was subsequently ligated into the linearized vector using T4 DNA ligase. The ligation products were transformed into *Escherichia coli* DH5α competent cells. The plasmids were extracted following antibiotic selection and amplification of bacterial cells. Successful construction of the recombinant plasmids was validated by Sanger sequencing.

### 2.16 Statistical analysis

All experiments were conducted independently at least in triplicate, and the value of n specifically denotes independent biological replicates rather than technical replicates. Data are presented as the mean ± standard deviation (SD). Statistical analyses were performed using GraphPad Prism 9.0 and SPSS 22.0 software. Prior to significance testing, data distribution normality was verified utilizing the Shapiro-Wilk test, and the homogeneity of variances was confirmed via Levene’s test. For data meeting these assumptions, statistical differences between two groups were evaluated using an unpaired Student’s t-test. Comparisons among three or more groups were analyzed using a one-way analysis of variance (ANOVA), followed by Tukey’s *post hoc* test for multiple comparisons across all groups, or Dunnett’s *post hoc* test when comparing multiple treatment groups against a single control group. Additionally, for data involving sequential measurements over time (e.g., *in vivo* tumor volume), a repeated measures two-way ANOVA was applied. Differences were considered statistically significant at *P* < 0.05.

## 3 Results

### 3.1 Gyp LI modulates autophagy-associated apoptosis in RCC cells

769-P and ACHN cell viability was assessed by CCK-8 assay following Gyp LI exposure for 24 h. The half-maximal inhibitory concentrations (IC50) after Gyp LI treatment for 24 h were 62.57 and 79.30 μM for 769-P and ACHN cells, respectively (Figure 2A). Next, the clonogenic and proliferative potential of the cells was evaluated by the colony formation assay. The overall colony-forming capacity substantially declined following Gyp LI treatment (Figure 2B). Subsequent fluorescence microscopy with Hoechst 33258 indicated that these specific interventions modulated the classical morphologic hallmarks of apoptosis, primarily characterized by nuclear disintegration and chromatin compaction (Figure 2C). The wound healing assay also indicated that Gyp LI remarkably inhibited the migratory potential of RCC cells at 24 h post-treatment (Figure 2D). Considering the biological effects observed in RCC cells after Gyp LI treatment and the critical role of autophagy in antitumor processes, we further hypothesized that autophagy may be involved in Gyp LI’s effect on RCC cells. The autophagic morphology of the cells was observed by TEM. Gyp LI treatment markedly increased the number of autophagosomes and autolysosomes in RCC cells, indicating characteristic autophagic changes (Figure 2E). The expression levels of autophagy-related proteins were quantified by RT-PCR and WB assay. The results showed increased ATG5, LC3 I/II, and Beclin1 expression and decreased P62 expression (Figures 2F,G). The expression levels of apoptosis-related proteins were estimated by WB assay. Bax and caspase-3 (proapoptotic proteins) showed increased expression, whereas Bcl-2 (antiapoptotic protein) exhibited decreased expression (Figure 2H). Collectively, these findings indicate that Gyp LI suppresses ccRCC, a process accompanied by apoptosis and molecular alterations in the autophagic pathway in RCC cells.

**FIGURE 2:**
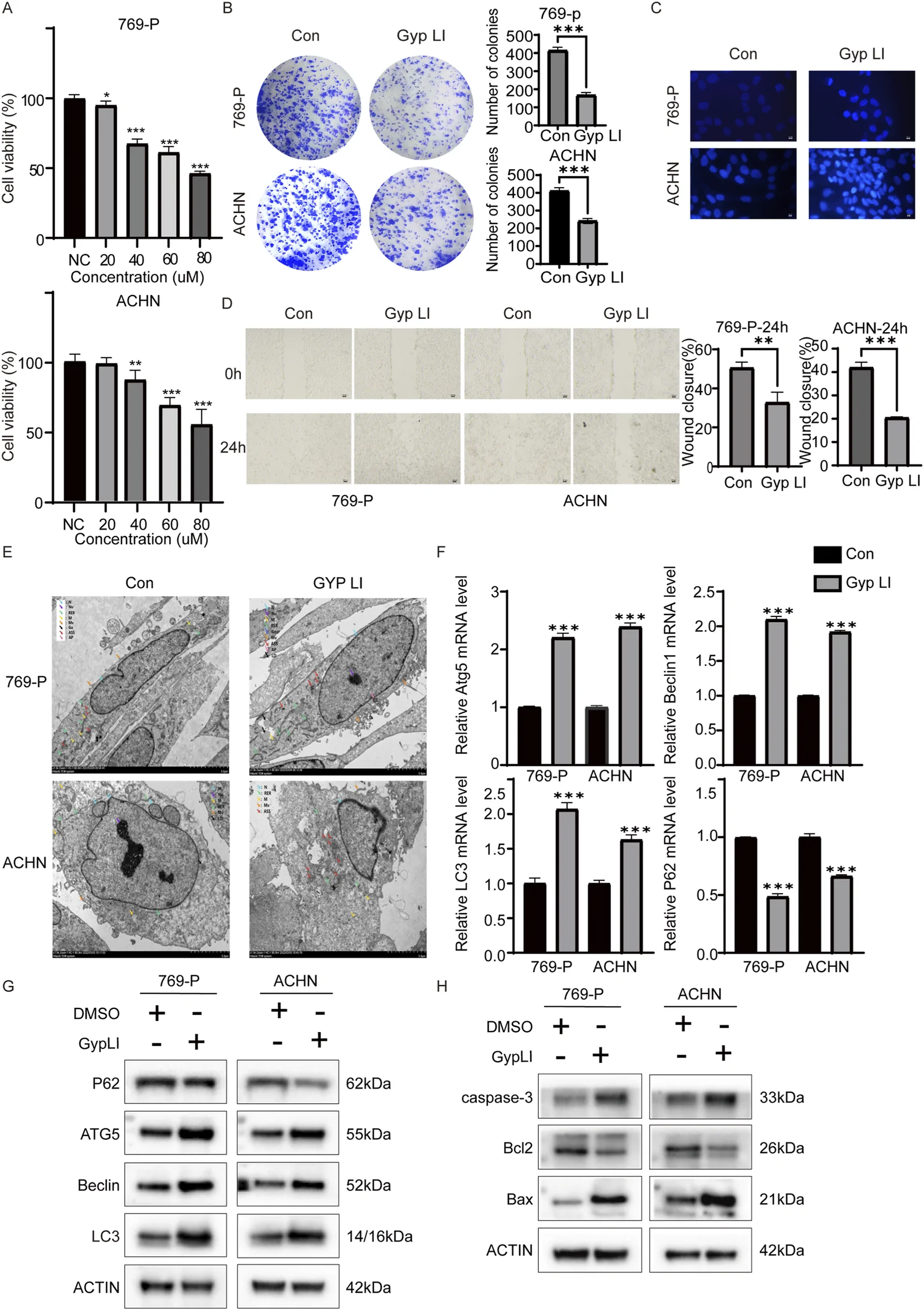
Gyp LI modulates autophagy-associated apoptosis in RCC cells. **(A)** Cell viability of 769-P and ACHN cells after 24 h of Gyp LI treatment, as detected by CCK-8 assay. **(B)** The clonogenic potential of RCC cells following Gyp LI treatment was determined by colony formation assay. **(C)** Morphological changes in cell nuclei of RCC cells exposed to Gyp LI for 24 h were observed by Hoechst 33258 staining. Representative indicators of apoptosis, such as fragmented nuclei paired with highly condensed chromatin, are clearly shown. **(D)** Wound healing assay for evaluating the migratory potential of RCC cells at 0 and 24 h post-treatment with Gyp LI. **(E)** Transmission electron microscopy images showing autophagic structures in 769-P and ACHN cells following Gyp LI treatment. The images show nuclei (N), nucleoli (Nu), rough endoplasmic reticulum (RER), mitochondria (M), microvilli (Mv), Golgi apparatus (Go), and autophagy-related structures [ASS: autophagic sequestration structures; AP: autophagosomes]. Arrows of different colors indicate the corresponding organelles and structures. **(F)** mRNA levels of autophagy-related genes in 769-P and ACHN cells following Gyp LI treatment. **(G)** Western blotting (WB) assay of Gyp LI’s effects on the expression of key autophagy markers, including LC3, P62, ATG5 and Beclin. **(H)** WB assay for quantifying expression of Bcl-2, Bax, and caspase-3 (apoptosis-related proteins) after 24 h of Gyp LI treatment. All data are presented as the mean ± SD from *n* = 3 independent biological replicates. Statistical significance was determined using one-way ANOVA followed by Dunnett’s *post hoc* test (for multiple comparisons against the control) or an unpaired Student’s t-test (for two-group comparisons). **P < 0.01 and ***P < 0.001 vs. the control group.

### 3.2 Gyp LI promotes phosphorylation-mediated ULK1-associated signaling

Unc-51-like autophagy activating kinase 1 (ULK1), a critical regulatory enzyme, serves as an indispensable molecular switch regulating the autophagic cascade. ULK1 is associated with autophagy-related Atg proteins to form the autophagy initiation complex, promotes type I to type II conversion of the autophagy marker LC3, and facilitates autophagosome formation (Hurley and Young, 2017). RT-qPCR showed that, compared to the control group, Gyp LI-treated 769-P and ACHN cells exhibited an increase in ULK1 mRNA levels (Figure 3A); however, WB assay showed no significant change in total ULK1 protein expression (Figure 3B). This discrepancy could be attributed to limited translational efficiency, accelerated protein degradation, or differences in detection time points. WB assay also demonstrated a marked increase in ULK1 phosphorylation (Figure 3B), suggesting that Gyp LI may regulate ULK1 mainly at the post-transcriptional level by enhancing ULK1 phosphorylation and thereby activating ULK1. It is important to note that the functional consequence of ULK1 phosphorylation is highly site-dependent. While phosphorylation at specific sites (such as Ser758) by mTOR exerts an inhibitory effect, the phosphorylation at Ser556 (corresponding to murine Ser555)—which was measured in this study—is widely recognized as a key regulatory modification closely associated with autophagy-related ULK1 signaling (Egan et al., 2011). Therefore, the increased phosphorylation of ULK1 at Ser556 observed upon Gyp LI treatment is consistent with enhanced ULK1-associated signaling, suggesting the engagement of this axis in the modulation of the autophagic process. Rescue experiments were then conducted for confirming the key role of ULK1 in Gyp LI-modulated autophagy. ULK1 was knocked down in 769-P and ACHN cells, followed by Gyp LI treatment. TEM showed that, compared to the abundant autophagosome formation observed in the Gyp LI-only group, autophagosome formation was reduced after ULK1 knockdown (Figure 3C). Consistently, analysis of autophagy-related proteins revealed that Gyp LI-modulated upregulation of ATG5, Beclin, and LC3-II/LC3-I was attenuated, whereas the abnormal change in P62 was also normalized (Figure 3D). Gyp LI modulates autophagic processes alongside the phosphorylation-mediated modulation of ULK1, and this phosphorylation event is closely associated with the autophagic alterations and the overall antitumor effects of Gyp LI.

**FIGURE 3:**
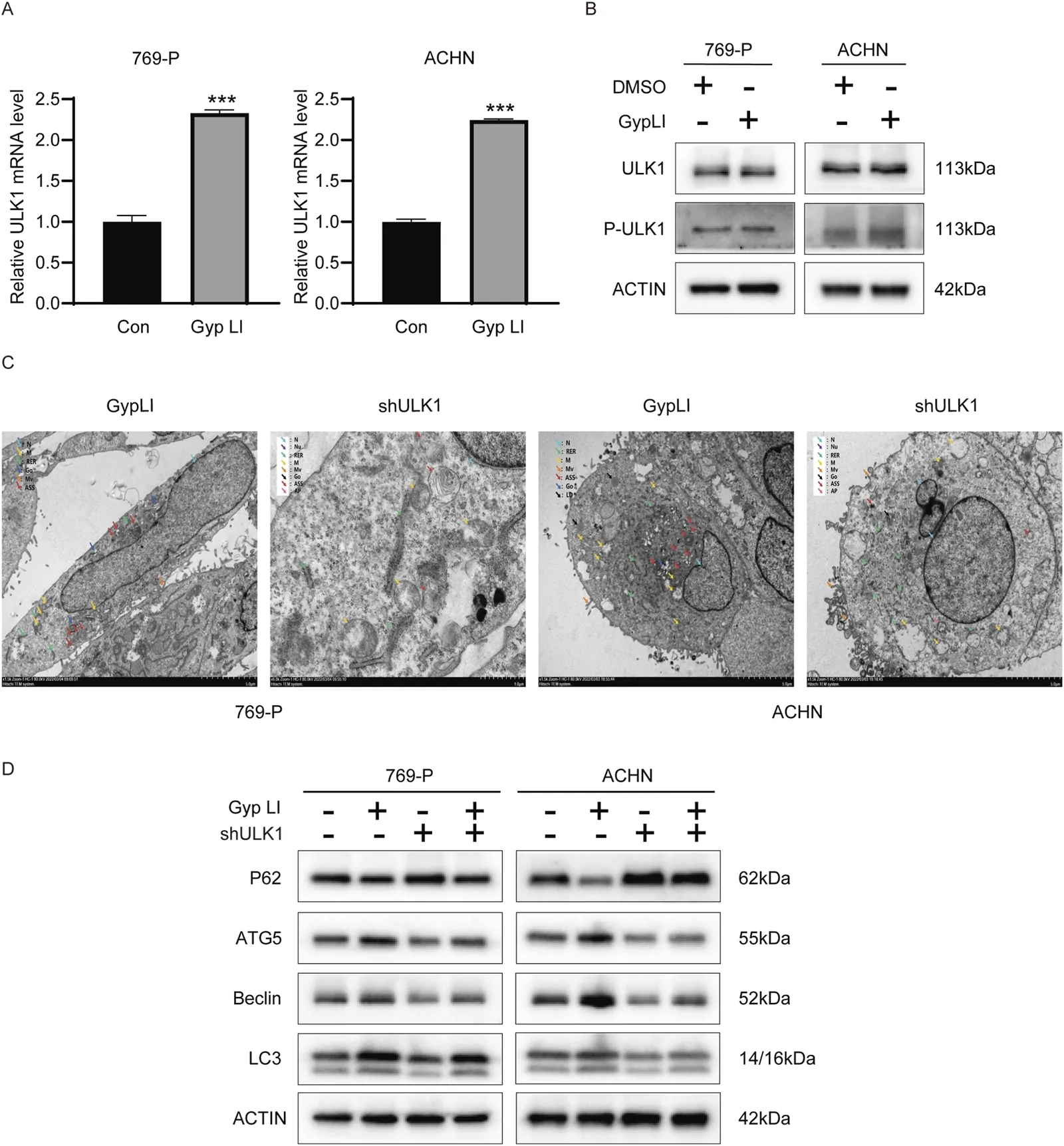
Gyp LI promotes phosphorylation-mediated modulation of ULK1. **(A)** ULK1 mRNA levels in 769-P and ACHN cells following treatment with Gyp LI. **(B)** Protein levels of ULK1 and P-ULK1 in Gyp LI-treated 769-P and ACHN cells. **(C)** TEM images showing autophagic structures in 769-P and ACHN cells. The images show nuclei (N), nucleoli (Nu), rough endoplasmic reticulum (RER), mitochondria (M), microvilli (Mv), Golgi apparatus (Go), and autophagy-related structures [ASS: autophagic sequestration structures; AP: autophagosomes]. Arrows of different colors indicate the corresponding organelles and structures. **(D)** WB assay showing the expression levels of the autophagy markers LC3, P62, ATG5 and Beclin. All data are presented as the mean ± SD from *n* = 3 independent biological replicates. Statistical significance was determined using an unpaired Student’s t-test (for two-group comparisons) or one-way ANOVA followed by Dunnett’s *post hoc* test (for multiple comparisons). *P < 0.05, **P < 0.01, and ***P < 0.001 vs. the control group.

### 3.3 Gyp LI modulates the transcription factor PPARG to promote PTEN expression

To facilitate PPI network construction and hub-target screening, the molecular structure of Gyp LI was drawn using ChemDraw and imported into the Swiss Target Prediction platform for target prediction. This database yielded 99 potential targets of Gyp LI. Furthermore, 3,684 RCC-related targets were retrieved from the GeneCards, OMIM, TTD, DrugBank, and PharmGKB databases. In the GeneCards database, the Relevance Score threshold was fixed at score ≥21.7 to control the number of targets, ensure credible associations, and standardize the screening criteria. A total of 57 common genes were identified by comparing Gyp LI-associated targets with RCC-related targets (Figure 4A). These 57 intersecting targets were then imported into the STRING database for constructing a PPI network, which finally included 56 proteins and 418 edges (Figure 4B). The CytoNCA plugin in Cytoscape v3.9.1 was used for topological analysis, and the structural importance of each node was evaluated according to the degree value. The results suggested that the transcription factor PPARG may be a core candidate regulator through which Gyp LI exerts its anti-ccRCC effect. Further validation in 769-P and ACHN cells showed that Gyp LI treatment substantially elevated PPARG mRNA and protein expression levels. PPARG, a nuclear receptor transcription factor, can regulate PTEN expression, while PTEN is a key negative regulator of the PI3K/AKT/mTOR signaling pathway (Han et al., 2024). Therefore, we further examined changes in PTEN expression in 769-P and ACHN cells. RT-qPCR and WB assay showed that Gyp LI treatment significantly upregulated PTEN mRNA and protein expression levels (Figure 4C). To elucidate the transcriptional regulatory relationship between PPARG and PTEN, ChIP-qPCR was conducted to analyze the chromatin-binding sites of PPARG. Significant enrichment of PPARG was observed in the PTEN promoter region (Figure 4D). The dual-luciferase reporter assay confirmed substantial enhancement of PTEN promoter activity by PPARG, indicating that PPARG can directly bind to and transcriptionally activate PTEN (Figure 4E). Next, we assessed changes in the key molecules of the PI3K/AKT/mTOR pathway and found that, following Gyp LI treatment, the PI3K/AKT/mTOR signaling axis was globally inhibited in 769-P and ACHN cells, with declining trends in the total expression and phosphorylation levels of the related proteins (Figure 4F). These findings suggest that Gyp LI upregulates PPARG expression, enhances PTEN transcriptional activity, increases PTEN expression, and inhibits the PI3K/AKT/mTOR signaling pathway, providing a molecular basis for its antitumor effect.

**FIGURE 4:**
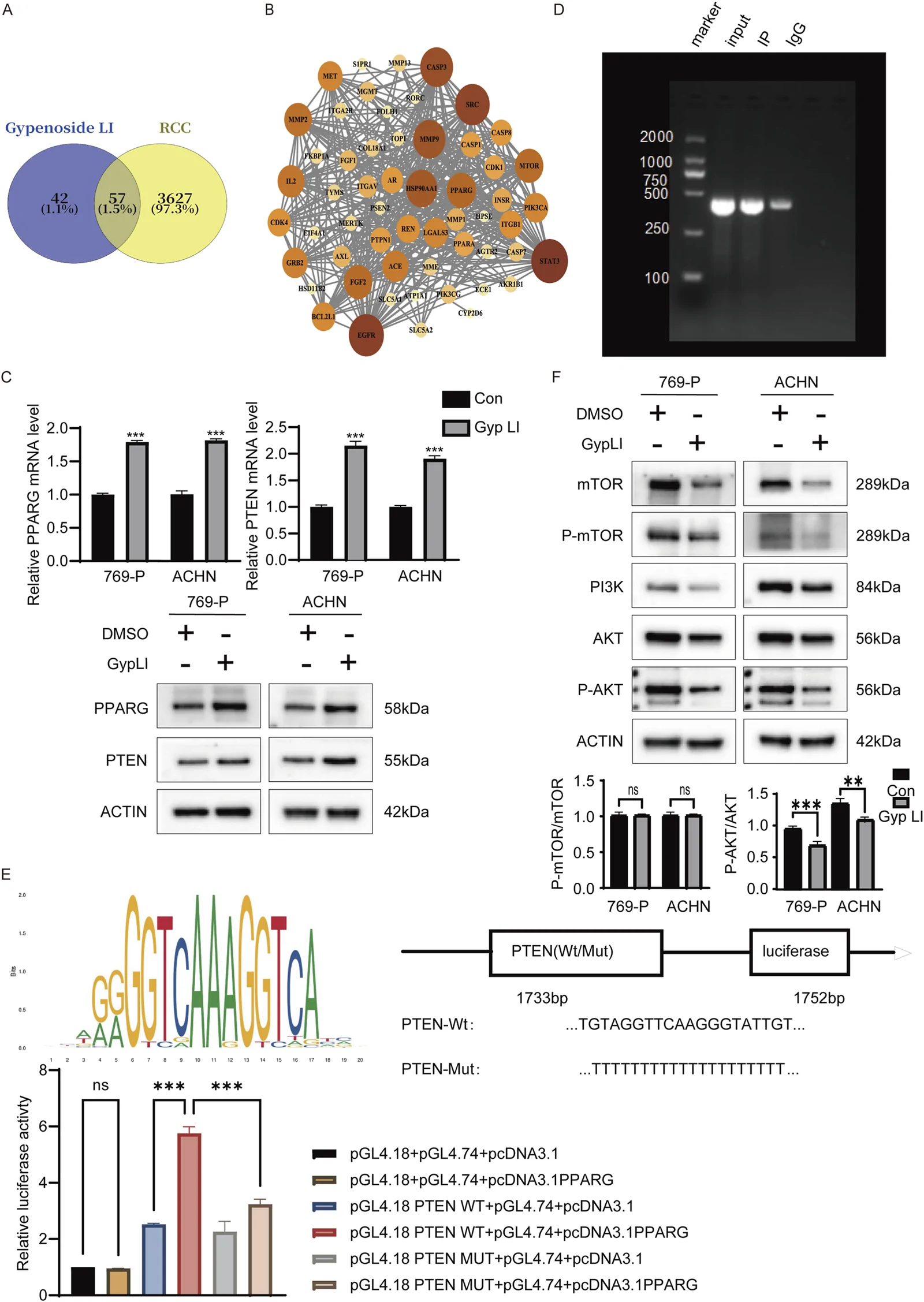
Gyp LI modulates the transcription factor PPARG to promote PTEN expression. **(A)** Network pharmacology analysis of potential candidate regulators of Gyp LI. **(B)** PPI network constructed for the intersecting targets. **(C)** PPARG and PTEN mRNA and protein expression levels in 769-P and ACHN cells after Gyp LI treatment. **(D)** ChIP-qPCR analysis of PPARG chromatin-binding sites and analysis of PTEN expression levels. **(E)** Dual-luciferase reporter assay evaluating PPARG’s effect on PTEN promoter activity. **(F)** Protein expression levels of mTOR, p-mTOR, PI3K, AKT, and p-AKT in 769-P and ACHN cells after Gyp LI treatment. To accurately assess pathway activity, the activation status is quantified and presented as p-AKT/Total-AKT and p-mTOR/Total-mTOR ratios. All data are presented as the mean ± SD from *n* = 3 independent biological replicates. Statistical significance was determined using an unpaired Student’s t-test (for two-group comparisons) or one-way ANOVA followed by an appropriate *post hoc* test (for multiple comparisons). ns no significant difference; **P < 0.01, and ***P < 0.001 vs. the control group.

### 3.4 Gyp LI modulates the PPARG-PTEN-PI3K/AKT/mTOR-ULK1 signaling axis

To confirm the mechanism by which Gyp LI mediates ULK1-associated signaling and modulates autophagy-associated apoptosis through the PPARG-PTEN-PI3K/AKT/mTOR-ULK1 signaling axis, we conducted *in vitro* rescue experiments in 769-P and ACHN cells. Colony formation assays revealed a distinct gradient in the clonogenic potential of 769-P and ACHN cells across the groups. The control group exhibited the highest number of colonies, followed by the shPPARG alone group and the shPPARG + Gyp LI combination group, whereas the Gyp LI alone group displayed the fewest colonies (Figure 5A). These results indicate that Gyp LI monotherapy exerts the strongest inhibitory effect on the clonogenicity of ccRCC cells, and PPARG knockdown partially attenuates this robust inhibition. Wound healing assays revealed a consistent trend: Gyp LI alone most significantly suppressed the wound closure rate in both cell lines, while the combined treatment partially restored the migratory capacity compared to the Gyp LI alone group (Figure 5B). Similarly, Hoechst 33258 staining showed that Gyp LI alone modulated the most prominent apoptotic features such as chromatin condensation and nuclear fragmentation. Conversely, PPARG knockdown reduced the proportion of apoptotic cells in the combination group compared to that in the Gyp LI monotherapy group (Figure 5C). Collectively, these phenotypic observations suggest that the anti-proliferative, anti-migratory, and proapoptotic effects of Gyp LI on RCC cells are closely associated with the functional status of PPARG, highlighting it as a relevant component in Gyp LI’s antitumor mechanism. Mechanistic investigations further substantiated this conclusion. As observed earlier, Gyp LI treatment significantly upregulated ULK1 phosphorylation level; however, this Gyp LI-modulated elevation in p-ULK1 was reversed following PPARG knockdown (Figure 5D), indicating that the phosphorylation and modulation of ULK1 are regulated by upstream PPARG signaling. Consistent with these signaling alterations, further phenotypic evaluations demonstrated that the Gyp LI-modulated autophagic phenotype was attenuated under PPARG-knockdown conditions (Figure 5E), and the aberrant expression trends of autophagy-related proteins were reversed and approached control levels (Figure 5F). Collectively, these findings suggest that PPARG not only mediates autophagy activation but also serves as a candidate regulator for Gyp LI-modulated cell death (including both apoptosis and autophagy) in RCC cells. These results suggest that Gyp LI modulates PPARG to promote the transcriptional upregulation of PTEN. This cascade associated with the PI3K/AKT/mTOR pathway, which is associated with the relief of ULK1 inhibition and its subsequent phosphorylation-mediated activation, ultimately contributing to the modulation of autophagy and tumor cell apoptosis.

**FIGURE 5:**
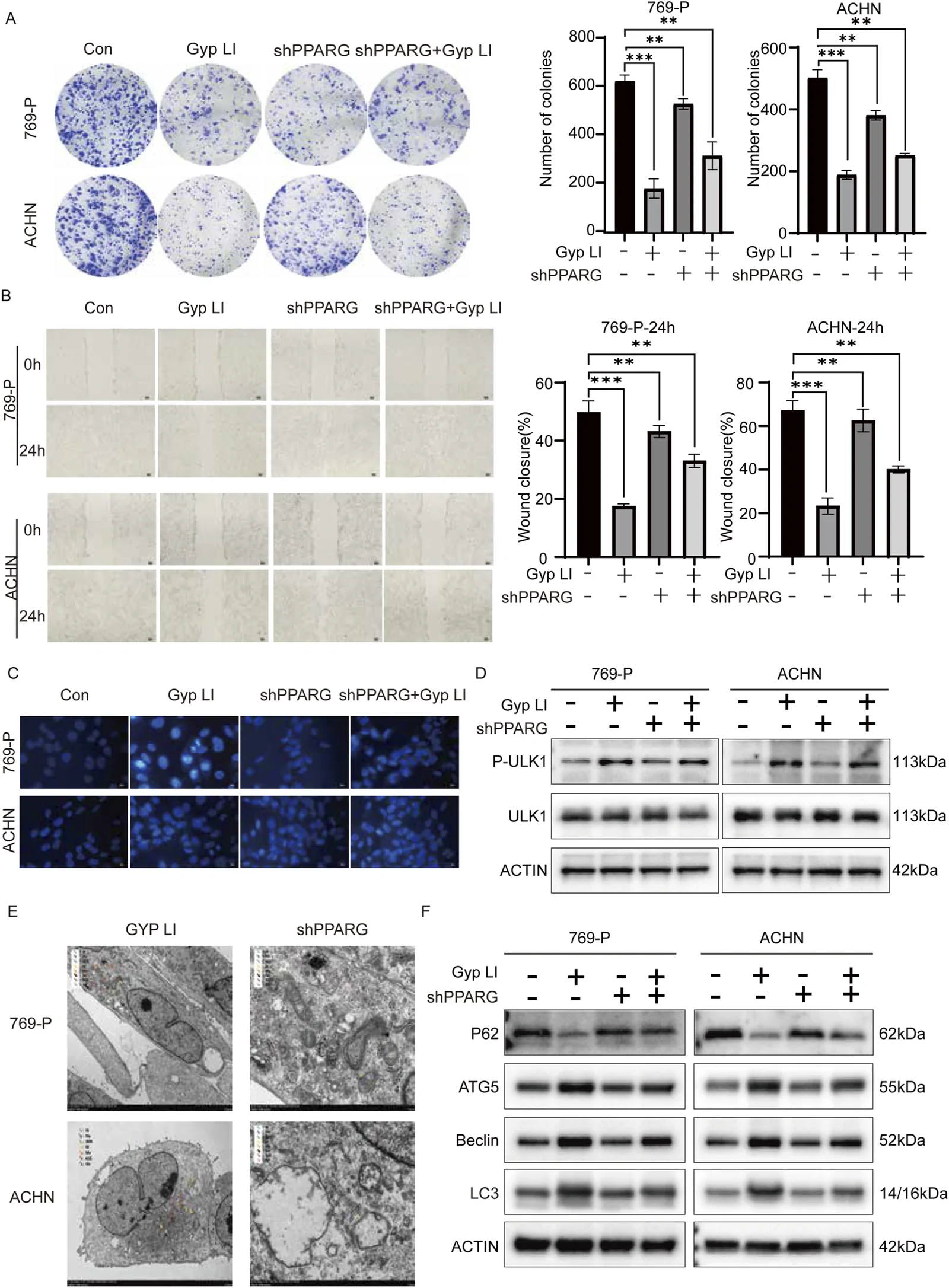
Gyp LI modulates the PPARG-PTEN-PI3K/AKT/mTOR-ULK1 signaling axis. **(A)** Colony formation assay assessing the clonogenic potential of 769-P and ACHN cells treated with shPPARG or Gyp LI, either alone or in combination. **(B)** Wound healing assay evaluating the migratory potential of cells across different treatment groups. **(C)** Hoechst 33258 staining illustrating the nuclear morphological changes and apoptotic features modulated by single or combined treatments. **(D)** WB assay of p-ULK1 expression after PPARG knockdown followed by Gyp LI treatment. **(E)** TEM images showing autophagic structures in Gyp LI-treated 769-P and ACHN cells under PPARG knockdown conditions. **(F)** WB assay of the effects of PPARG knockdown on the expression levels of key autophagy markers, including LC3, P62, ATG5 and Beclin All data are presented as the mean ± SD from *n* = 3 independent biological replicates. Statistical significance was determined using one-way ANOVA followed by Tukey’s *post hoc* test for multiple comparisons among all indicated groups. *P < 0.05, **P < 0.01, and ***P < 0.001.

### 3.5 Gyp LI suppresses ccRCC growth *in vivo*

A xenograft model was established for evaluating the inhibitory effect of Gyp LI on renal cancer progression *in vivo*. Briefly, 5 × 10^6^ ACHN cells were subcutaneously implanted into the right region behind the scapula of BALB/c nude mice. When tumor volume became 100 mm^3^, tumor-bearing mice were assigned randomly to four groups (4 mice per group): shCon group, shPPARG group, shPPARG + Gyp LI group and Gyp LI group. Tumor volume was recorded at 4-day intervals. Tumor tissues were collected after 35 days of treatment. The Gyp LI and shPPARG + Gyp LI groups exhibited tumor-inhibitory effects, with the Gyp LI group exhibiting the most prominent inhibitory effect (Figures 6A–C). Moreover, mice treated with Gyp LI and/or knockdown showed stable body weight (Figure 6D). Hematoxylin and eosin staining of liver and kidney samples revealed no significant structural differences, indicating no apparent hepatotoxicity or nephrotoxicity under the tested experimental conditions (Figure 6E). IHC staining further showed that, compared to the shPPARG group, the Gyp LI and shPPARG + Gyp LI groups exhibited upregulated ATG5, Beclin1, Bax, and caspase-3 expression and downregulated Bcl-2 and Ki-67 expression. These changes were more prominent in the shPPARG + Gyp LI and Gyp LI groups, suggesting that Gyp LI modulated autophagy and apoptosis and inhibited tumor cell proliferation in ACHN xenograft tissues (Figure 6F). TUNEL staining further showed few apoptotic cells in the shCon group; however, apoptotic signals were enhanced in the combined treatment group, with Gyp LI treatment exhibiting the most prominent apoptotic effect (Figure 6G). WB assay showed that Gyp LI treatment functionally suppressed the PI3K/AKT/mTOR signaling pathway, as indicated by decreased PI3K levels and increased expression of PTEN and ULK1. The Gyp LI treatment group exhibited the strongest effect (Figure 6H). Gyp LI decreased total AKT protein abundance and reduced the p-AKT/Total-AKT ratio, suggesting a functional link between AKT suppression and PPARG. Concurrently, mTOR total protein abundance also decreased, whereas its relative phosphorylation level (p-mTOR/Total-mTOR ratio) remained unchanged, indicating that Gyp LI impairs the mTOR axis primarily through total protein depletion. These results suggest that Gyp LI promotes autophagy and apoptosis in ACHN cells and suppresses tumor growth by modulating PPARG and inhibiting the PI3K/AKT/mTOR signaling pathway.

**FIGURE 6:**
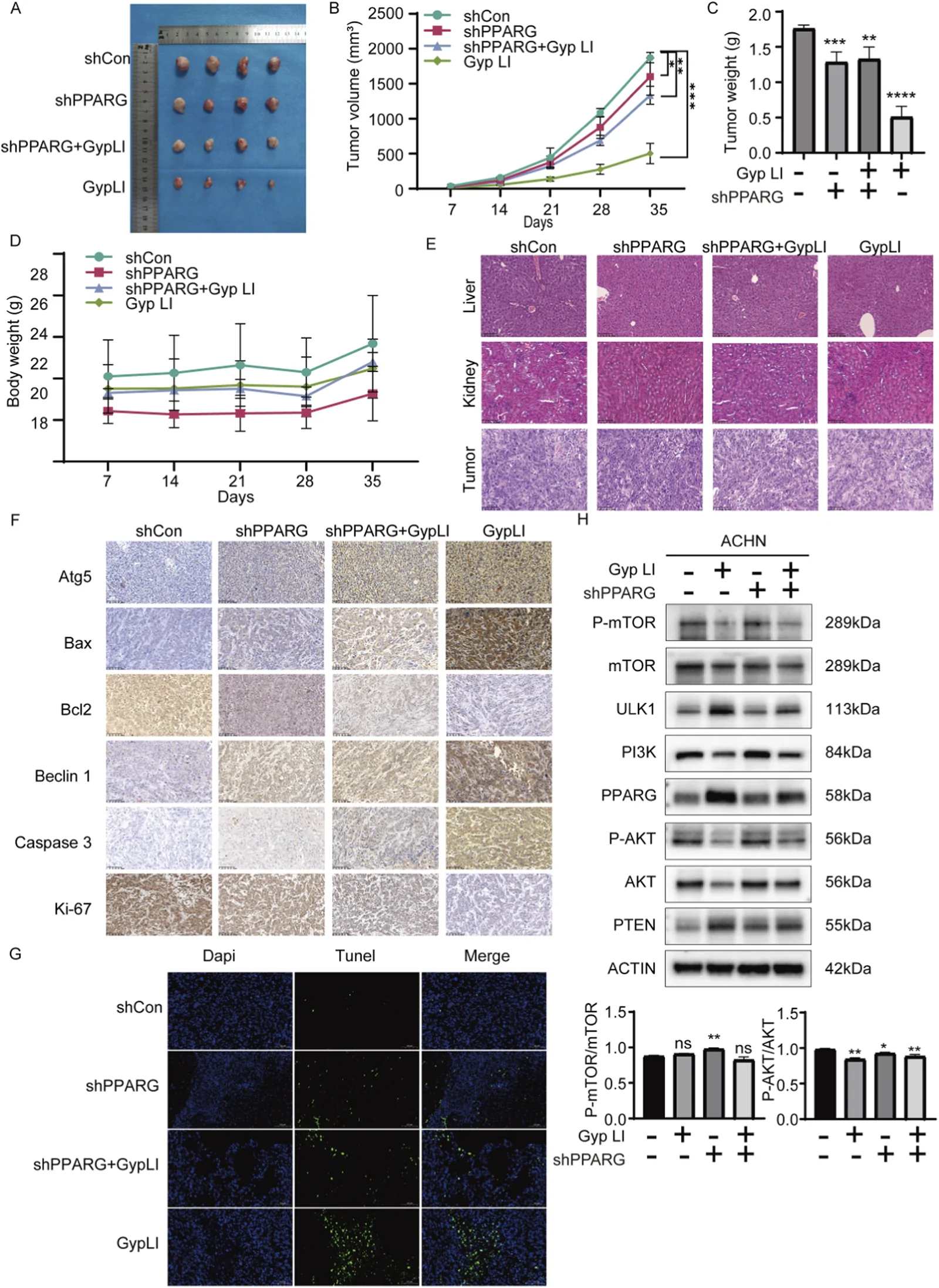
Gyp LI inhibits RCC cell growth *in vivo* and regulates autophagy-related pathways. **(A)** Representative images of excised tumors at the observation endpoint. **(B)** Growth curves of nude mouse xenografts after the indicated treatments (*n* = 4). **(C)** Statistical analysis of tumor weight (*n* = 4). **(D)** Statistical analysis of nude mouse body weight (*n* = 4). **(E)** Hematoxylin and eosin staining of liver and kidney tissues, exhibited no apparent toxicity under the tested experimental conditions. **(F)** Immunohistochemical detection of ATG5 Bax, Bcl-2, Beclin 1, caspase-3, and Ki-67 expression in xenograft tumor tissues from each group. **(G)** TUNEL staining to detect apoptosis in xenograft tumor tissues from each group. Nuclei are stained by DAPI (blue), TUNEL-positive cells appear green, and Merge indicates merged images. **(H)** WB assay of p-mTOR, mTOR, ULK1, PI3K, PPARG, p-AKT, AKT, PTEN, and ACTIN protein expression in xenograft tumor tissues from each group. The pathway activation status within the complex *in vivo* microenvironment is stringently quantified and presented as p-AKT/Total-AKT and p-mTOR/Total-mTOR ratios. All data are presented as the mean ± SD from n = 4 independent animals per group. Statistical significance for tumor growth curves **(B)** was determined using a repeated measures two-way ANOVA. **(C)** Tumor weight endpoint data were analyzed by one-way ANOVA with Dunnett’s *post hoc test*. **(D)** Body weight changes were analyzed by repeated measures two-way ANOVA. ns no significant difference; *P < 0.05, **P < 0.01, ***P < 0.001, and ****P < 0.0001 vs. the shCon group (or vehicle control).

## 4 Discussion

RCC presents a critical challenge in oncology in terms of early diagnosis and precision treatment because of its strong invasiveness, frequent recurrence, high metastatic potential, and poor prognosis (Rini et al., 2009). Despite continuous advancements in therapeutic approaches for RCC, notable limitations persist, underscoring the need to comprehensively investigate its complex biology and design novel therapeutic strategies (Cooley et al., 2021). The pathogenesis of ccRCC extends far beyond basic morphological alterations, involving a highly complex network of molecular dysregulations and targeted therapeutic resistance. Recent high-quality studies have provided profound mechanistic insights into ccRCC progression. For instance, Wang et al. recently demonstrated that Trigonostemon xyphophyllorides exerts significant anti-proliferative effects against renal cell carcinoma by modulating the PI3K-AKT signaling pathway (Wang et al., 2025). Similarly, another pivotal study highlighted that oxymatrine suppresses the progression of renal cell carcinoma both *in vitro* and *in vivo* by upregulating TOR1AIP1 expression. This upregulation in turn inactivates the JNK signaling pathway, which otherwise regulates renal tumor advancement and therapeutic vulnerability (Yan et al., 2025). The present study contributes to meaningful progression in understanding RCC and designing effective interventions for its treatment.

As a prominent bioactive saponin derived from *Gynostemma pentaphyllum*, Gypenoside LI has been historically recognized for its pharmacological safety and efficacy in regulating lipid metabolism, attenuating inflammation, and ameliorating metabolic syndromes (Su et al., 2021; Liu et al., 2022). In recent years, its application has increasingly expanded into the field of oncology. Emerging evidence integrating network pharmacology and *in vitro* assays indicates that total gypenosides can modulate tumor cell apoptosis by modulating the PI3K/AKT/mTOR signaling pathway, thereby exhibiting significant inhibitory effects against various solid tumors, including renal, gastric, and bladder carcinomas (Liu et al., 2021; Li et al., 2022; Wu et al., 2024). *G. pentaphyllum* and total gypenosides have been studied for metabolic and inflammatory disorders; however, evidence specifically concerning purified Gypenoside LI remains limited.

At the molecular level, the PI3K/AKT/mTOR signaling axis is frequently aberrantly activated in ccRCC and shows a close association with tumor cell proliferation, survival, invasion, and therapeutic resistance (Fan et al., 2020; Jin et al., 2023; Hsieh et al., 2017). *PTEN*, a tumor suppressor gene and a classical negative regulator of this pathway, can promote tumor progression following a reduction in its expression or impairment of its function (Song et al., 2012; Bu et al., 2021). Autophagy has also been widely studied as a therapeutic target in multiple diseases because of its involvement in disease progression (Tan et al., 2017). Autophagy has a dual role in ccRCC. Under diverse genetic backgrounds, metabolic states, and therapeutic pressures, autophagy may help tumor cells adapt to adverse environments by eliminating damaged organelles and maintaining energy homeostasis, or it may lead to cell death and enhanced antitumor effects when strongly or persistently activated (Levy et al., 2017; White, 2015). Mechanistically, Gyp LI regulates this balance by inhibiting AKT/mTOR signaling (Hu et al., 2026). Therefore, achieving effective regulation of the intensity and direction of autophagy in specific contexts is critical to optimize therapeutic strategies for ccRCC.

ULK1 is the primary kinase of the autophagy initiation complex and converts nutrient and energy status signals into autophagy initiation events (Kim et al., 2011). In the canonical regulatory axis, mTOR1 inhibits autophagy by phosphorylation and suppression of ULK1, whereas AMPK can directly phosphorylate and activate ULK1 to promote autophagy initiation (Wang et al., 2022). In RCC, although autophagy may help tumor cells survive hypoxia, nutrient deprivation, or therapeutic stress, it may also trigger cell death under certain conditions. Thus, ULK1 often reflects the dependence of tumor cells on stress adaptation and metabolic maintenance (Rebecca and Amaravadi, 2016). Notably, previous studies have shown that upregulated ULK1 in ccRCC is associated with a poor prognosis; moreover, ULK1 knockdown can modulate apoptosis in ccRCC cells and affect metabolic flux by blocking autophagy, suggesting that ULK1 may represent a functional vulnerability in ccRCC(Lu et al., 2018). In the present study, Gyp LI modulates autophagy by promoting phosphorylation-mediated modulation of ULK1, demonstrating that ULK1 phosphorylation is an important molecular event through which Gyp LI modulates autophagy and exerts antitumor effects.

PTEN, a key negative regulator of the PI3K-AKT-mTOR pathway, can enhance PI3K/AKT signaling tone and promote tumor progression following its loss or inactivation (Miricescu et al., 2021); therefore, PTEN alterations are considered relevant to ccRCC occurrence, progression, and treatment response. PPARG is a nuclear receptor transcription factor associated with lipid metabolism, differentiation, and inflammation (Ahmadian et al., 2013). From a mechanistic network perspective, PTEN downregulation usually enhances the PI3K-AKT-mTOR signaling axis, thereby affecting cellular anabolism, survival, and migration. In RCC cells, increased PTEN expression can inhibit the PI3K/AKT signaling pathway and reduce cell viability and migration, indicating that PTEN suppresses the biological characteristics of RCC(Xu et al., 2025). PPARG may indirectly regulate PI3K signaling flux by transcriptionally controlling lipid metabolism- and inflammation-related genes, thereby altering upstream growth factor signaling. Previous studies have also proposed that the tumor-suppressive effects of PPARG agonists may be mediated by PTEN upregulation (Burge et al., 2025; Chen et al., 2024), providing biological feasibility for a functional connection along the PPARG-PTEN-PI3K/AKT axis. In the present study, Gyp LI treatment activated the transcription factor PPARG. Moreover, PPARG knockdown reversed Gyp LI’s effects on cell proliferation, autophagy, and apoptosis. One limitation of the initial target screening in this study is the reliance on a single prediction platform (Swiss Target Prediction), which theoretically carries a risk of coincidental findings. However, our mechanistic interpretation does not rely solely on the Swiss Target Prediction result. Our results showed that Gyp LI upregulated PPARG expression and promoted its binding to the PTEN promoter, thereby enhancing PTEN transcription and inhibiting the PI3K/AKT/mTOR signaling axis, which subsequently promoted phosphorylation-mediated modulation of ULK1, modulates autophagy, and induced apoptosis. Rescue experiments using PPARG knockdown reversed the increase in p-ULK1 and the related phenotypes, further supporting a causal association within this signaling axis. Based on these observations, we propose that Gyp LI regulates autophagy-associated apoptosis in ccRCC cells by upregulating PPARG and coordinately modulating the PTEN/PI3K/AKT/mTOR-ULK1 signaling network.

This study has several limitations. First, despite the observation of significant autophagic alterations, autophagic flux was not directly assessed. Subsequent studies employing an mRFP-GFP-LC3 reporter, pharmacological inhibitors, and genetic knockdown are required to confirm autophagic flux activation and determine whether Gyp LI-modulated apoptosis is strictly autophagy-dependent. Second, while Gyp LI was shown to modulate PPARG signaling, their direct physical interaction remains elusive, necessitating further validation using advanced target-binding assays. Specifically, PTEN acts as a critical intermediate node in this proposed signaling network. Since direct PTEN knockdown or specific pharmacological manipulation was not performed in the current study, we cannot definitively rule out the possibility of parallel regulatory mechanisms bypassing PTEN. Future investigations utilizing targeted PTEN silencing or specific inhibitors are imperative to explicitly map the epistatic dependencies and fully validate this sequential signaling cascade. Third, the lack of toxicity evaluation on normal human cell lines is a limitation. Future studies are required to systematically investigate its safety profile and potential side effects. Furthermore, regarding the *in vivo* experiments, the relatively small sample size (*n* = 4 per group) and the lack of investigator blinding during measurements represents methodological limitations, which may constrain the statistical power. In addition, stable body weights and normal liver/kidney histology do not establish comprehensive biosafety. Future studies involving larger cohorts, rigorous blinding, and extensive systemic toxicological profiling are required to fully ascertain the safety and translational potential of Gypenoside LI. In the present study, 769-P and ACHN cell lines were utilized as the primary *in vitro* models because they are widely used RCC/ccRCC-related experimental models, providing a reliable foundation for our mechanistic explorations. While consistent therapeutic effects of Gypenoside LI were observed in both cell lines, the lack of validation across a broader panel of ccRCC cell lines remains a limitation. Future studies will be conducted to evaluate its efficacy in other ccRCC cell models to further confirm the universality of its anti-tumor effects.

## 5 Conclusion

This study demonstrates that Gyp LI possesses significant anti-ccRCC activity both *in vitro* and *in vivo*, without apparent *in vivo* toxicity. Mechanistically, Gyp LI upregulates PTEN expression via the modulation of PPARG; these cascades associate with the functional inhibition of the PI3K/AKT/mTOR signaling pathway, correlating with the phosphorylation-associated modulation of ULK1. While Gyp LI was shown to modulate PPARG signaling, their direct physical interaction remains elusive, necessitating further validation using advanced target-binding assays. Ultimately, these events modulate autophagy-associated apoptosis in ccRCC cells. In conclusion, these findings provide preclinical evidence supporting the anti-tumor potential of Gyp LI in ccRCC models. However, further comprehensive studies are warranted, as the current work does not yet establish its clinical efficacy or safety profile.

## Data Availability

The datasets presented in this study can be found in online repositories. The names of the repository/repositories and accession number(s) can be found in the article/supplementary material.
